# Novel Transcatheter Edge-to-Edge Repair System for Degenerative Mitral Regurgitation: 1-Year Outcomes

**DOI:** 10.1016/j.jacasi.2026.04.013

**Published:** 2026-06-12

**Authors:** Da Zhu, Zhenfei Fang, Xianbao Liu, Nan Zhang, Yuhao Liu, Yan Wang, Liang Ma, Jinjun Liu, Jian Yang, Yan Li, Caidong Luo, Xiaoping Peng, Wei Zhong, Liangwan Chen, Jian Xu, Fan Ouyang, Jiancheng Xiu, Jinping Liu, Ben He, Mao Chen, Shenghua Zhou, Xiangbin Pan

**Affiliations:** a Structural Heart Center, Fuwai Yunnan Hospital, Chinese Academy of Medical Sciences, Affiliated Cardiovascular Hospital of Kunming Medical University, Kunming, China; b Department of Cardiology, The Second Xiangya Hospital of Central South University, Changsha, China; c Department of Cardiology, The Second Affiliated Hospital, Zhejiang University School of Medicine, Zhejiang, China; d Department of Cardiology, Fuwai Central China Cardiovascular Hospital, Zhengzhou, China; e Department of Cardiology, Xiamen Cardiovascular Hospital Xiamen University, Xiamen, China; f Department of Cardiovascular Surgery, The First Affiliated Hospital, Zhejiang University School of Medicine, Hangzhou, China; g Department of Cardiology, The First Affiliated Hospital of Bengbu Medical University, Anhui, China; h Department of Cardiovascular Surgery, The First Affiliated Hospital of Air Force Military University, Xian, China; i Department of Cardiology, The Second Affiliated Hospital of Air Force Military University, Xian, China; j Department of Cardiology, Mianyang Central Hospital, Mianyang, China; k Department of Cardiology, The First Affiliated Hospital of Nanchang University, Nanchang, China; l Department of Cardiology, Meizhou People's Hospital, Meizhou, China; m Department of Cardiovascular Surgery, Fujian Medical University Union Hospital, Fujian, China; n Department of Cardiology, Anhui Provincial Hospital, Anhui, China; o Department of Cardiology, Nanfang Hospital, Guangzhou, China; p Department of Cardiovascular Surgery, Zhongnan Hospital of Wuhan University, Wuhan, China; q Department of Cardiology, Shanghai Chest Hospital, Shanghai, China; r Department of Cardiology, West China Hospital Sichuan University, Chengdu, China; s Structural Heart Center, Fuwai Hospital, Chinese Academy of Medical Sciences, Beijing, China

**Keywords:** degenerative mitral regurgitation, GeminiOne, transcatheter edge-to-edge repair

## Abstract

**Background:**

For patients with severe degenerative mitral regurgitation (DMR) at prohibitive or high surgical risk, transcatheter edge-to-edge repair (TEER) has become an effective alternative. The GeminiOne is a novel TEER device featuring a unique groove-cam mechanism.

**Objectives:**

This study reports the primary, secondary, and 1-year outcomes of the GeminiOne-DMR (Safety and Efficacy of the GeminiOne Transcatheter Valve Edge-to-Edge Repair System in Patients With Moderate-severe or Severe Degenerative Mitral Regurgitation) trial.

**Methods:**

A total of 120 patients with prohibitive surgical risk and DMR grade ≥3+ were enrolled and evaluated by an independent echocardiography core laboratory and a clinical event committee. The primary endpoint was clinical success, defined as freedom from all-cause mortality, mitral valve reintervention, and mitral regurgitation >2+ at 1-year follow-up.

**Results:**

At 1 year, the clinical success rate was 89.84% ± 2.78% (95% CI: 84.56%-95.46%), meeting the prespecified primary efficacy endpoint. The rates of freedom from 1-year all-cause mortality, major adverse events, mitral valve reintervention, and heart failure hospitalization were 97.48% ± 1.35%, 91.61% ± 2.54%, 98.31% ± 1.02%, and 94.00% ± 2.20%, respectively. Mitral regurgitation ≤2+ was achieved in 92.24% of patients at 1 year. Significant improvements were observed in functional and quality-of-life outcomes, with the proportion of patients classified as NYHA funcional class I/II increasing from 23.33% at baseline to 94.74% at 1 year (*P* < 0.001), and Kansas City Cardiomyopathy Questionnaire scores also improving significantly from baseline to 1 year (*P* < 0.001).

**Conclusions:**

This trial demonstrated the initial safety and efficacy of the GeminiOne TEER system for treating significant symptomatic DMR in patients at prohibitive or high surgical risk. (Safety and Efficacy of the GeminiOne Transcatheter Valve Edge-to-Edge Repair System in Patients With Moderate-severe or Severe Degenerative Mitral Regurgitation; NCT05655897)

Mitral regurgitation (MR) is one of the most prevalent valvular heart diseases worldwide.^1^^,^^2^ The mitral valve transcatheter edge-to-edge repair (TEER) has become an important, guideline-recommended treatment option for patients deemed unsuitable for surgery including degenerative mitral regurgitation (DMR) and functional mitral regurgitation (FMR).^3^^,^^4^ For patients with DMR, the Food and Drug Administration has approved 2 types of TEER devices: the PASCAL transcatheter valve repair system (Edwards Lifesciences) and the MitraClip system (Abbott Vascular).

The GeminiOne transcatheter edge-to-edge repair system (Peijia Medical) is conceptually similar to the MitraClip and PASCAL systems but features a unique, novel clip design that incorporates an innovative groove-cam mechanism. The GeminiOne-DMR (Safety and Efficacy of the GeminiOne Transcatheter Valve Edge-to-Edge Repair System in Patients With Moderate-severe or Severe Degenerative Mitral Regurgitation) trial aims to evaluate the safety and efficacy of the GeminiOne system in patients experiencing symptomatic moderate-to-severe and severe DMR who are considered to be at high surgical risk and whose mitral anatomy is suitable for the TEER procedure.

## Methods

### Trial design and patient selection

This prospective, multicenter, single-arm study enrolled clinically symptomatic patients with chronic moderate-to-severe (3+) or severe (4+) DMR who were deemed to be at high surgical risk or unsuitable for conventional surgery by a multidisciplinary heart team, including at least 1 cardiac surgeon and 1 cardiologist. High surgical risk was defined as a Society of Thoracic Surgeons (STS) score of ≥8 for valve replacement, or ≥6 for valve repair, or the presence of 2 or more indicators of frailty. Eligible participants were ≥18 years old and categorized in NYHA functional class II, III, or ambulatory class IV. Echocardiographic inclusion criteria included a left ventricular end-systolic diameter ≤60 mm, a mitral valve orifice area ≥4.0 cm^2^, and a left ventricular ejection fraction ≥20%. Anatomical suitability was assessed independently by a designated eligibility committee.

Key exclusion criteria included a history of mitral valve surgery; evidence of an intracardiac mass, thrombus, or infective endocarditis; the presence of other severe non–mitral valve conditions requiring intervention such as untreated severe coronary artery disease; recent acute myocardial infarction, transient ischemic attack, or stroke occurring within the past 30 days; pulmonary hypertension (pulmonary artery systolic pressure >70 mm Hg); a life expectancy <12 months; any cardiovascular interventional procedure performed within 30 days; and calcification of the mitral valve leaflets at the grasping zones. The complete inclusion and exclusion criteria are available in Supplemental Table 1.

Follow-up visits were conducted immediately after the procedure; before discharge; and at 1, 6, and 12 months post procedure. All echocardiograms were evaluated by an independent core laboratory, and any major adverse events (MAEs) were reviewed by an independent clinical events committee. The study protocol was approved by local ethics committees at each research site including ethics committees of Fuwai Yunnan Hospital (principal investigation site, Approval Number: 2022-049-02). Informed written consent was obtained from all patients. The trial was conducted in accordance with Good Clinical Practice principles and the Declaration of Helsinki. The study protocol was designed in alignment with the guidelines established by the Mitral Valve Academic Research Consortium.^5^ Trial organization and participating sites are listed in Supplemental Table 2. The trial was registered at ClinicalTrials.gov (NCT05655897).

### Study device

**Figure 1:**
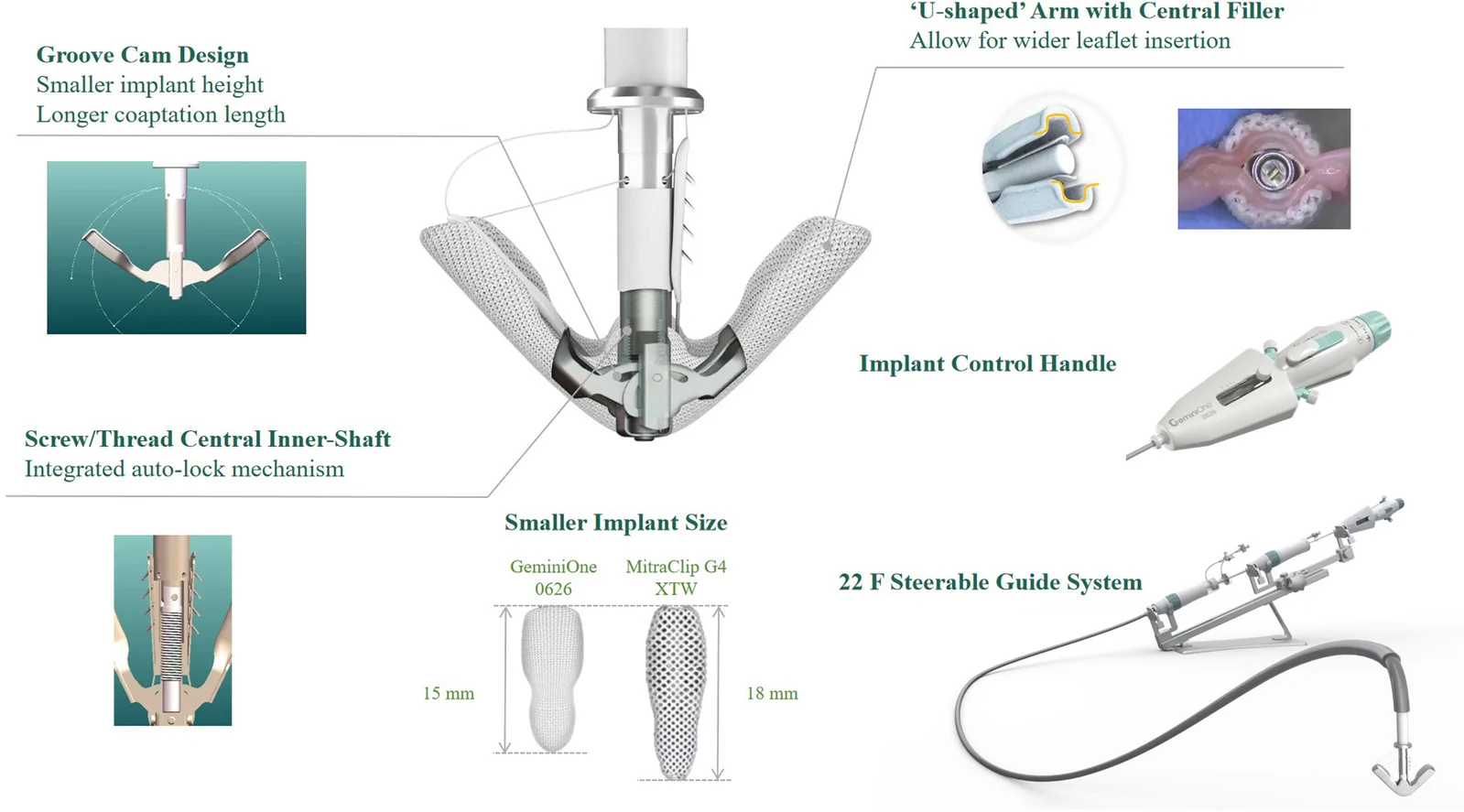
Design of GeminiOne System The system has 4 different clip sizes (4/6 mm in clip arm width, 10/13 mm in clip arm length) with an independent leaflet grasping design. A 22-French steerable guide catheter was used with a 16-French transcatheter edge-to-edge repair delivery system. The innovative groove-cam mechanism allows for automatic locking and release of the clip at any angle. A “U-shape” arm with a threaded central shaft structure allows for wider leaflet insertion. The clip length in the closed position is also shortened compared with the MitraClip and, therefore, requires less supravalvular height for delivery and positioning.

The GeminiOne system (Peijia Medical Limited) is a novel designed TEER system, classified as a fourth-generation TEER system (Figure 1). A total of 4 different clip sizes (4/6 mm in clip arm width, 10/13 mm in clip arm length) with independent leaflet grasping are strategically designed to accommodate different mitral valve pathologies. The system employs a 22-French steerable guide catheter, along with a 16-French TEER delivery system. The unique feature of the GeminiOne system is the innovative groove-cam mechanism combined with a threaded central shaft structure (Video 1). This design allows for automatic locking and release of the clip at any angle, thereby enabling the operator to adjust the clipping force and balance the leaflet tension during the procedure. In addition, due to the unique groove-cam locking mechanism, no locking maneuver is necessary during clip implantation. The clip length in the closed position is shorter compared with the MitraClip, necessitating less supravalvular height for delivery and positioning. Moreover, the maximum clip capture length (06/26 size: 6-mm arm width and 13-mm arm length) reaches 25 mm at 120 degrees and 26 mm at 180 degrees, even surpassing the capabilities of the MitraClip G4 system with XTW clip.^6^ The implantation procedure has been previously described.^6^

### Trial endpoints

The primary endpoint was the clinical success at 12 months, defined as freedom from all-cause mortality, the necessity for re-intervention due to mitral valve dysfunction, and residual MR >2+. Secondary endpoints included technical success immediately after the procedure, device success at 30 days, MR ≤2+ rates at 1 year, and improvements in functional status evaluated by NYHA functional class and Kansas City Cardiomyopathy Questionnaire (KCCQ) score at 1 year. Technical success was defined as successful deployment and leaflet grasping, followed by the retrieval of the delivery system, without death or emergency surgery related to the device or procedure. Device success was defined as freedom from all-cause mortality, stroke, the need for re-intervention due to mitral valve dysfunction, residual MR >2+, and no significant mitral stenosis.

Major adverse events (MAEs) were evaluated through 1 year and comprised all-cause mortality, stroke, myocardial infarction, major bleeding, renal failure requiring renal replacement therapy, and nonelective cardiovascular surgery or reintervention due to device or procedure-related adverse events, such as single leaflet device attachment (SLDA). Safety endpoints encompassed all-cause mortality; cardiovascular death; and MAE rates at 1, 6, and 12 months. Heart failure hospitalization (HFH) was an extended observational endpoint. Procedure-induced mitral stenosis and other clinical complications were noted in the study cohort.

### Echocardiographic assessments

Both transthoracic echocardiography and transesophageal echocardiography were performed at local sites in accordance with acquisition protocols established by the independent core laboratory, primarily for patient screening and procedural planning. Follow-up transthoracic echocardiography evaluations were analyzed by the core laboratory, using evaluation criteria based on the 2017 Recommendations for Noninvasive Evaluation of Native Valvular Regurgitation by the American Society of Echocardiography, 2022 Multi-modality imaging assessment of native valvular regurgitation by the European Association of Cardiovascular Imaging, and the European Society of Cardiology. MR severity was graded on a scale ranging from 0 to 4+.

### Statistical analysis

This prospective, multicenter, single-arm trial was designed to evaluate the efficacy and safety of the GeminiOne system, using a predefined performance goal. The sample size calculation was based on the primary efficacy endpoint, defined as the treatment success rate at 12 months after the procedure. Assuming a historical clinical event rate of 60%^7^, ^8^, ^9^ and considering a maximum potential dropout rate of 10%, it was estimated that enrolling 120 patients would provide 80% power to detect a significant difference. The treatment group was expected to achieve a 12-month postprocedural treatment success rate of 73%.

Data on demographics, clinical characteristics, echocardiography, medical therapy, and surgery were presented as numbers and percentages for categorical variables, and as mean ± SD or medians (Q1, Q3) for continuous variables. Paired Student's *t*-tests were used for continuous variables including KCCQ score and echocardiographic metrics (eg, mitral effective orifice area), and McNemar tests were applied for categorical variables including NYHA functional class and degree of MR, to compare changes from baseline to 1, 3, 6, and 12 months of follow-up. The annualized rate of MAEs (including mortality, reintervention due to mitral dysfunction, MR >2+, HFH, and renal failure) was estimated using the Kaplan-Meier method with censoring applied for patients without events or lost to follow-up. The standard error was calculated using Greenwood’s formula, and intergroup comparisons were performed using the log-rank test, with the corresponding *P* value calculated. A 2-sided *P* value of <0.05 was considered statistically significant. All statistical analyses were performed using R software, version 4.4.1.

## Results

### Study population

From November 2022 to May 2024, a total of 120 patients from 19 sites were enrolled in this study. The 1-year follow-up was completed in May 2025. All patients underwent the TEER procedure and were included in the full analysis set. The mean age of the study population was 72.95 ± 4.94 years, with 48.3% (58 of 120) female participants, and the mean body mass index was 22.69 ± 3.09. In total, 34.2% (41 of 120) of patients had been hospitalized for heart failure (HFH) within 1 year before the TEER procedure. Median Society of Thoracic Surgeons score for replacement of the study cohort was 8.40 (6.76, 8.94). At baseline, 76.7% (92 of 120) of patients were classified as NYHA functional class ≥III and 37.5% (45 of 120) exhibited ≥2 moderate-to-severe indicators of frailty. Baseline demographics and comorbidities of subjects are presented in Table 1. All patients had an MR grade ≥3+, with 91.67% (110 of 120) having an MR grade >4+. In terms of valve pathology, 62.5% (75 of 120) had prolapse or flail involving the A2/P2 area, whereas 37.5% (45 of 120) presented with prolapse or flail in the non-A2/P2 area. Detailed echocardiographic data are shown in Table 2.

**Table 1:** Baseline Characteristics (N = 120)

Demographics	
Age, y	72.95 ± 4.94 (120)
Female	48.3 (58/120)
Height, cm	161.03 ± 8.14 (120)
Weight, kg	58.93 ± 9.63 kg (120)
BMI, kg/m^2^	22.69 ± 3.09
STS score for mitral valve replacement	8.40 (6.76,8.94)
STS score for mitral valve repair	4.90 (3.36,6.73)
NYHA functional class	
II	23.3 (28/120)
III	62.5 (75/120)
IV	14.2 (17/120)
KCCQ score, point	77.61 (61.25,85.89)
Medical history/comorbidity	
Hypertension	59.2 (71/120)
Diabetes	15.8 (19/120)
Smoke	25.0 (30/120)
Prior hospitalization for heart failure within l year	34.2 (41/120)
Previous PCI	15 (18/120)
Previous stroke or TlA	11.7 (14/120)
Peripheral vascular diseases	25.0 (30/120)
Pulmonary hypertension	20.8 (25/120)
Chronic kidney disease	20.0 (24/120)
Coronary heart disease	43.3 (52/120)
MI	17.3 (9/52)
Previous heart surgery	1.7 (2/120)
Transcatheter aortic valve intervention	0.8 (1/120)
Chronic obstructive pulmonary disease	9.2 (11/120)
Modified Rankin score	0.71 ± 1.20 (14)
Pacemaker implantation	0 (0/120)
ICD	0 (0/120)

Values are mean ± SD (N), median (Q1, Q3) or % (n/N).

BMI = body mass index; ICD = implantable cardioverter-defibrillators; KCCQ = Kansas City Cardiomyopathy Questionnaire; MI = myocardial infarction; PCI = percutaneous coronary intervention; STS = Society of Thoracic Surgeons; TIA = transient ischemic attack.

**Table 2:** Baseline Echocardiographic Measures (N = 120)

Degenerative mitral regurgitation etiology	86.7 (104/120)
Mixed MR etiology	13.3 (16/120)
LVEF %	62.35 (55.80,67.88)
MR grade	
0	0 (0/120)
1+	0 (0/120)
2+	0 (0/120)
3+	8.33 (10/120)
4+	91.67 (110/120)
EROA, cm^2^	0.52 (0.39,0.75)
RV, mL	71.00 (51.00,100)
TAPSE, mm	18.38 ± 5.26 (93)
MVOA, cm^2^	6.39 (5.58,7.47)
TMPG, mm Hg	2.74 (2.00,4.00)
PASP	36.97 ±13.04 (117)
Length of anterior leaflet, mm	23.59 ±3.94 (119)
Length of posterior leaflet, mm	13.87 ±3.08 (117)
MR mechanism	
Anterior prolapse/Flail	30.8 (37/120)
Posterior prolapse/Flail	68.3 (82/120)
Bileaflet prolapse	0.8 (1/120)
Prolapse/Flail width, mm	14.20 (11.20,16.00)
Prolapse/Flail gap, mm	4.65 (3.30,6.00)
Prolapse/Flail location, n (%)	
Posterior leaflet	
P2	53 (44.2)
Non-P2	29 (24.2)
Anterior leaflet	
A2	22 (18.3)
Non-A2	15 (12.5)
Bi-leaflet	
A3P3	1 (0.8)
LVESD, mm	32.00 (27.51,36.80)
LVEDD, mm	50.10 (44.90,54.35)
LVESV, mL	37.35 (26.50, 50.75)
LVEDV, mL	98.05 (73.67,131.70)
TR	
No regurgitation	21 (17.5)
1+	69 (57.5)
2+	14 (11.7)
3+	13 (10.8)
4+	3 (2.5)

Values are mean ± SD (N), median (Q1,Q3) or n (%).

DMR = degenerative mitral regurgitation; EROA = effective regurgitation orifice area; LVEDD = left ventricular end-diastolic diameter; LVEDV = left ventricular end-diastolic volume; LVEF = left ventricular ejection fraction; LVESD = left ventricular end-systolic diameter; LVESV = left ventricular end-systolic volume; MR = mitral regurgitation; MVOA = mitral valve orifice area; PASP = pulmonary artery systolic pressure; RV = regurgitation volume; TAPSE = tricuspid annular plane systolic excursion; TMPG = transmitral mean pressure gradient; TR = tricuspid regurgitation.

### Procedure outcomes

The technical success rate of the GeminiOne device implantation was 98.33% (118 of 120). One patient experienced an unsuccessful treatment due to a clip-related leaflet tear, leading to open heart surgery immediate after the procedure. Multiple lealflet grasping during operation and suboptimal loose tension were believed to be the main reasons for this complication. Another patient failed due to a suspected air embolism associated with the guiding catheter, which led to switching to the MitraClip device. The operator failed to follow the deairing process of the GeminiOne device in his first case. Device success at 30 days was achieved in 95.83% (115 of 120). The median time for device implantation was 72 minutes (54, 120). Among the patients, 1 GeminiOne device was successfully implanted in 53.3% (64 of 120), 2 devices in 45.0% (54 of 120), and 3 devices in 1 patient (0.8%, 1 of 120). Procedural details are shown in Table 3. A learning curve analysis indicated a slight reduction in device implantation time from the first stage (60 cases, median: 72 minutes, Q1, Q3: 57.5, 120, with a total of 87 clips implanted) to the second stage (60 cases, median: 68 minutes, Q1, Q3: 48.5, 120, with a total of 88 clips implanted).

**Table 3:** Procedural Data (N = 120)

Successful device implantation^a^	98.3% (118/120)
Number of devices implanted	
0	0.8% (1/120)
1	53.3% (64/120)
2	45.0% (54/120)
3	0.8% (1/120)
Number of devices implanted	1.46 ± 0.53 (120)
Size of device	100%(175)
FG22000-0620	7.4% (13/175)
FG22000-0420	8.0% (14/175)
FG22000-0626	74.3% (130/175)
FG22000-0426	10.3% (18/175)
Implantation location	
A1P1	13.7% (24/175)
A2P2	63.4% (111/175)
A3P3	22.9% (40/175)
Device implantation duration, min^b^	72 (54, 120)

Values are n (%), median (Q1, Q3), or mean ± SD (N).

Abbreviation as in Table 2.

a Successful implantation: successful delivery and deployment of 1 or more devices, confirmation of valve leaflet grasping by echocardiography, and successful withdrawal of the delivery system.

b Device implantation duration: from the introduction of the guide sheath into the femoral vein to its withdrawal from the femoral vein.

### Primary efficacy endpoint

**Figure 2:**
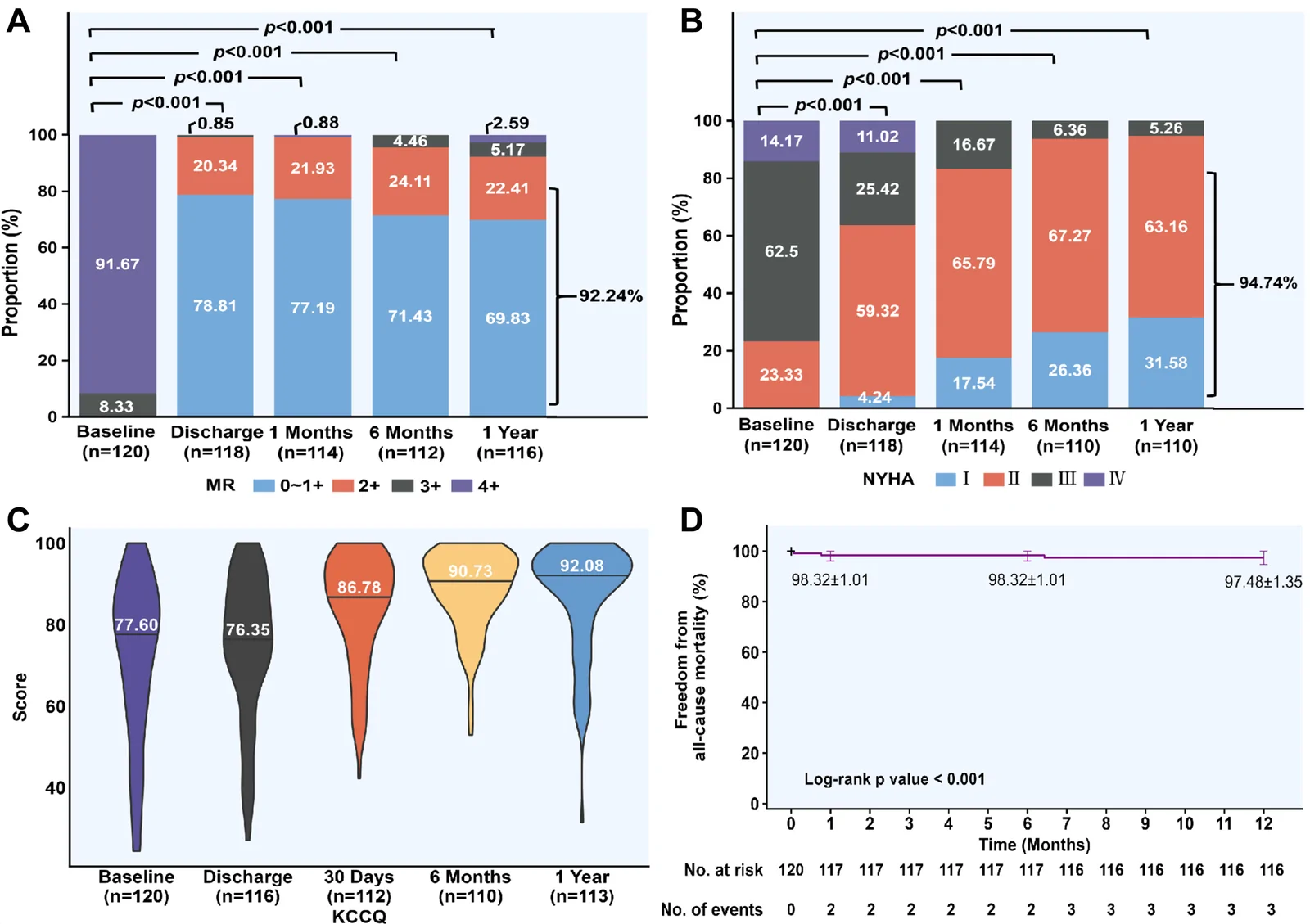
MR Degree, NYHA Functional Class, KCCQ Score, and Freedom From All-Cause Mortality at 1-Year Follow-Up (A) MR reduction assessed by the core laboratory before and after the procedure. (B) NYHA functional class improvement at 1 year. *P* values for intergroup comparisons of degree of MR and NYHA functional class were calculated using the McNemar test. (C) Improvement in KCCQ scores at 1 year, with data presented as medians. (D) Kaplan-Meier estimates for freedom from all-cause mortality (Kaplan-Meier estimate±SE). Error bars represent 95% CIs. KCCQ = Kansas City Cardiomyopathy Questionnaire; MR = mitral regurgitation.

Kaplan-Meier survival analysis was used to evaluate the full analysis set and demonstrated a clinical success rate of 89.84% ± 2.78% (95% CI: 84.56%-95.46%) at 1 year after the procedure. The lower bound of the 95% CI exceeds the prespecified performance goal, confirming that the primary endpoint was successfully met. All patients included in the baseline data presented with an MR grade ≥3+, as determined by the independent echocardiographic core laboratory. At discharge and during follow-ups at 30 days, 6 months, and 1 year, the proportion of patients with MR grade ≤2+ was 99.15%, 99.12%, 95.54%, and 92.24%, respectively (Figure 2). Compared with baseline, these improvements were statistically significant. For different lesion locations, the proportions of patients with MR ≤2+ at discharge, 30 days, 6 months, and 1 year were 100.0%, 100.0%, 93.1%, and 89.0% for those with prolapse or flail involving the A2/P2 area, whereas those without A2/P2 involvement showed proportions of 97.7%, 97.6%, 100.0%, and 97.7%, respectively. Left ventricular end-diastolic volume demonstrated a marked reduction from 98.05 (73.67-131.70) mL at baseline to 81.45 (65.80-106.25) mL at 1 year (paired change = 16.21 mL; *P* < 0.05).

### Safety endpoint

**Figure 3:**
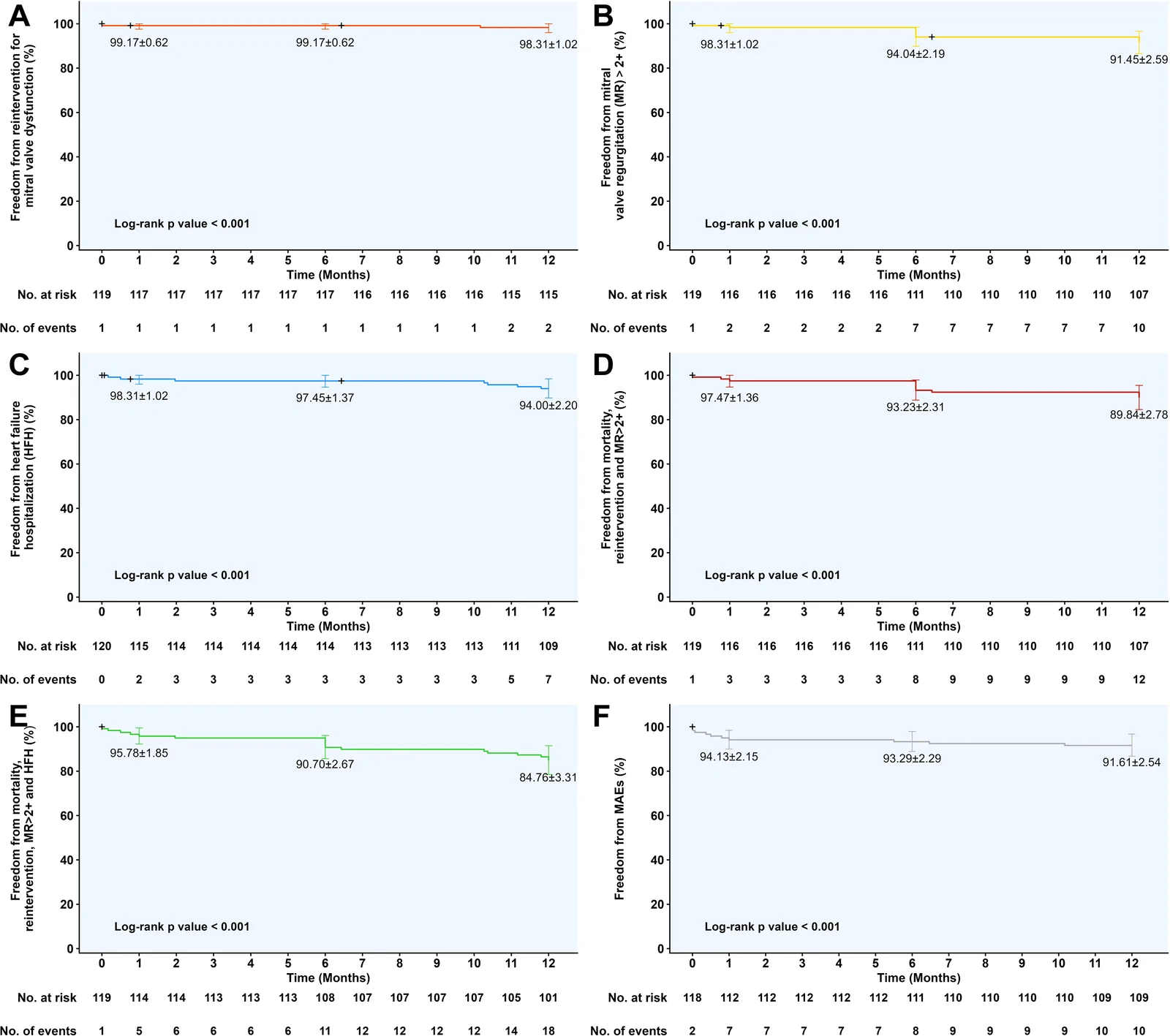
Freedom From Clinical Events Committee–Adjudicated Safety Endpoints The Kaplan-Meier estimates for freedom from (A) reintervention for mitral valve dysfunction, (B) MR >2+, (C) heart failure hospitalization (HFH), (D) clinical success at 1 year, and (E) composite of above events and (F) freedom from major adverse events (MAEs). The graph shows Kaplan-Meier estimate ± SE and error bars represent 95% CI. Plus symbols (+) indicate censored observations (lost to follow-up). Intergroup comparisons between 1 month and 1 year after intervention were performed using the log-rank test. Abbreviation as in Figure 2.

The Kaplan-Meier estimates for freedom from all-cause mortality, reintervention due to mitral valve dysfunction, mitral valve regurgitation >2+, HFH, and a composite of these events were 97.48% ± 1.35%, 98.31% ± 1.02%, 91.45% ± 2.59%, 94.00% ± 2.20%, and 84.76% ± 3.31%, respectively (Figures 2 and 3).

At 1 year, a total of 14 MAEs were reported across 10 patients, including 3 deaths (of which 2 were classified as cardiovascular mortality by the clinical events committee, including 1 open heart surgery–related death due to leaflet injury immediately after the TEER procedure, and 1 major stroke-related death at 6 months), another patient died due to postoperative major gastrointestinal bleeding, 3 ischemic strokes (1 occurred in the postoperative period and 2 were noted at 6 months’ follow-up), 3 major bleeding (both of them were gastrointestinal bleeding, 2 occurred in the postoperative period, and 1 was noted at 30 days’ follow-up), 2 renal failure (both occurred at 30 days’ follow-up), 2 reinterventions (1 open heart surgery due to clip-induced leaflet injury and 1 redo TEER procedure due to SLDA and significant MR at 1-year follow-up), and 1 acute myocardial infraction, which was noted at 6 months’ follow-up. Other complications included 1 case of acute pulmonary embolism and 1 case of DeBakey III aortic dissection; both occurred before discharge and were successfully treated. Seven patients were noted with HFH at the 1-year follow-up (Table 4). The 1-year Kaplan-Meier estimate for freedom from MAE was 91.61% ± 2.54% (Figure 3).

**Table 4:** Clinical Events Committee–Adjudicated Safety Endpoints at 1 Year (N = 120)

Major adverse events at 1 y	10 (8.39)
All-cause death	3 (2.52)
Stroke	3 (2.54)
Major bleeding	3 (2.54)
Nonelective mitral valve reintervention	2 (1.69)
Myocardial infraction	1 (0.85)
Renal failure	2 (1.72)
Other events at 1 y	
Heart failure hospitalization	7 (6.03)
Device-related air embolism	1 (0.83)
Pulmonary embolism	1 (0.85)
Aortic dissection	1 (0.85)

Values are n (%).

### Functional evaluation

At 12 months, the site-assessed NYHA functional class showed significant improvement. The percentage of patients categorized as class I/II increased from 23.33% at baseline to 94.74% at 12 months (*P* < 0.001), and the overall median KCCQ score increased from 77.60 at baseline to 92.08 at 1 year (Δ = 14.79; 95% CI:10.97-18.62; *P* < 0.001) (Figure 2).

## Discussion

MR is the most common valvular disease globally,^1^^,^^2^ with its incidence rising with age. Significant MR carries a 5-year mortality exceeding 20%,^10^ highlighting the need for timely treatment. Although surgical repair or replacement is the standard therapy for DMR, many patients are ineligible due to age, comorbidities, or high surgical risk.^3^^,^^4^ TEER has consistently demonstrated safety and efficacy^11^, ^12^, ^13^, ^14^, ^15^ and is approved in both Europe and the United States for high-risk patients with DMR and for symptomatic FMR patients despite guideline-directed medical therapy. The MitraClip and PASCAL are 2 commercially available TEER systems. In this prospective single-arm multicenter pivotal study, the novel GeminiOne TEER system demonstrated a favorable safety profile, an optimal procedural success rate, durable reduction of MR, and improvements in functional status and quality of life through 1 year. The study successfully achieved its prespecified primary efficacy endpoint, demonstrating clinical outcomes comparable to established devices such as MitraClip and PASCAL in the DMR population.

The incidence of MAEs remained low from 30 days to 1 year postprocedure. The 1-year freedom from MAEs rate was 91.61%, demonstrating a favorable safety profile for this novel TEER system. In addition, the 1-year rates for freedom from all-cause mortality and HFH (97.48% and 94.00%, respectively) were comparable to those reported in previous studies using classic TEER systems. In EVEREST II (Endovascular Valve Edge-to-Edge Repair Study II), the mortality rate at 1 year with the MitraClip device was 6%.^15^ In CLASP IID (Edwards PASCAL TrAnScatheter Valve RePair System Pivotal Clinical Trial), the Kaplan-Meier estimate rate for freedom from MAEs at 1 year was 84.7% for the PASCAL group and 88.3% for the MitraClip group.^11^ Corresponding 1-year Kaplan-Meier estimates for freedom from all-cause mortality were 91.2% for PASCAL and 91.6% for MitraClip, and the rates for freedom from HFH were 91.9% and 96.7%, respectively.^11^ In the DMR cohort of EXPAND G4 (MitraClip EXPAND G4 Study) using the fourth-generation MitraClip, the 1-year mortality rate was 8.4%, and the 1-year HFH rate was 10.4%.^12^ These results indicate that the GeminiOne TEER system achieved clinical outcomes that are comparable to those of established and mature TEER systems.

In terms of reducing MR, the GeminiOne TEER system achieved a 99.1% rate of MR reduction to ≤2+ at 30 days, which is comparable to the results obtained with the PASCAL (98.4%) and MitraClip (95.6%) devices in CLASP IID.^11^ At 1 year, 92.2% of patients maintained an MR ≤2+, surpassing the 81.7% reported in EVEREST II^15^ and comparable with the MitraClip (93.8%) and PASCAL (95.8%) outcomes from CLASP IID.^11^ Notably, 37.5% of patients in this study presented with lesions outside the A2/P2 region, indicating an increasing anatomical complexity. Further subgroup analysis revealed similar effectiveness in MR reduction for both the A2/P2 and non-A2/P2 regions, achieving MR ≤2+ at 1 year in 89.0% of A2P2 lesions and in 97.7% of non-A2P2 lesions. These results suggest that, even with limited experience with TEER procedures among Chinese operators, GeminiOne provides robust and durable regurgitation reduction across a variety of lesion locations.

Regarding quality of life and functional status, patients who underwent the TEER procedure using the GeminiOne system demonstrated substantial improvements. At 1 year, 94.7% of patients were classified as NYHA functional class I or II, which is slightly higher than the rates reported in CLASP IID (86.8% for MitraClip and 88.3% for PASCAL) and EXPAND G4 (82.1%). This suggests a favorable functional recovery.^11^^,^^15^ The mean KCCQ score increased by 14.79 at 1 year in the study cohort, similar to the improvements of 15.1 and 15.2 observed in the MitraClip and PASCAL subgroups of CLASP IID, respectively, and an 18.5 increase in EXPAND G4. These findings indicate that quality-of-life improvement associated with GeminiOne is comparable to that achieved by established TEER devices.^11^^,^^13^^,^^15^

The MitraClip and the PASCAL device are currently available mainstream TEER devices with different design concepts. For FMR, the MitraClip is commonly used because of its reliable mechanical locking design and robust closure force. For DMR, both devices have been proven effective. However, for selected patients with fragile leaflets or annular calcification, the PASCAL device may be a more suitable option because of its recoil-locking design. In contrast to the MitraClip and PASCAL, the GeminiOne system features a unique groove-cam locking mechanism and a U-shaped clip arm with a threaded central shaft. This innovative design allows for automatic locking and releasing of the clip at any angle, which enables the operator to adjust the clipping force and balance leaflet tension during the procedure. It effectively combines the advantages of both mechanical and recoil-based locking clips, providing sufficient closure force while protecting the leaflets and minimizing the risk of injury. As a novel TEER device, the GeminiOne system also prioritizes procedural ergonomics and operator usability. Features such as the elimination of repetitive lock–unlock maneuvers, a streamlined single-step release mechanism (without locker/Gripper line removal maneuver), and a window displaying the arm’s open angle at the clip control handle are all designed to reduce technical complexity and shorten the learning curve. In comparison to the Chinese-made DragonFly system,^16^ the device time for the GeminiOne system was even shorter (72 minutes vs 90 minutes). When comparing the first 60 cases with the subsequent 60 procedures, a slight reduction in device time was observed (72 minutes vs 68 minutes), indicating a steady learning curve. During the TEER procedure for DMR, complications such as SLDA and loss of leaflet insertion remain major concerns.^17^, ^18^, ^19^ These events can arise from inadequate leaflet grasping or damage to the native leaflet.^20^^,^^21^ Previous bench testing demonstrated that the U-shaped clip arm with a central shaft design allowed for broader leaflet insertion and provides a stronger anchoring force, potentially improving leaflet capture in DMR patients with excessive leaflet motion and redundant tissue.^22^ In addition, the GeminiOne system integrates features from the fourth-generation TEER system, including independent leaflet grasping and multiple clip sizes. Due to its groove-cam design, the clip is shorter in the closed position compared with the MitraClip, which reduces the required supravalvular height for delivery and positioning. Furthermore, when using the optimal leaflet-grasping angle of 120 degrees, its arm span is even larger than that of the MitraClip, which helps enhance leaflet capture effectiveness (Central Illustration).

**Central Illustration:**
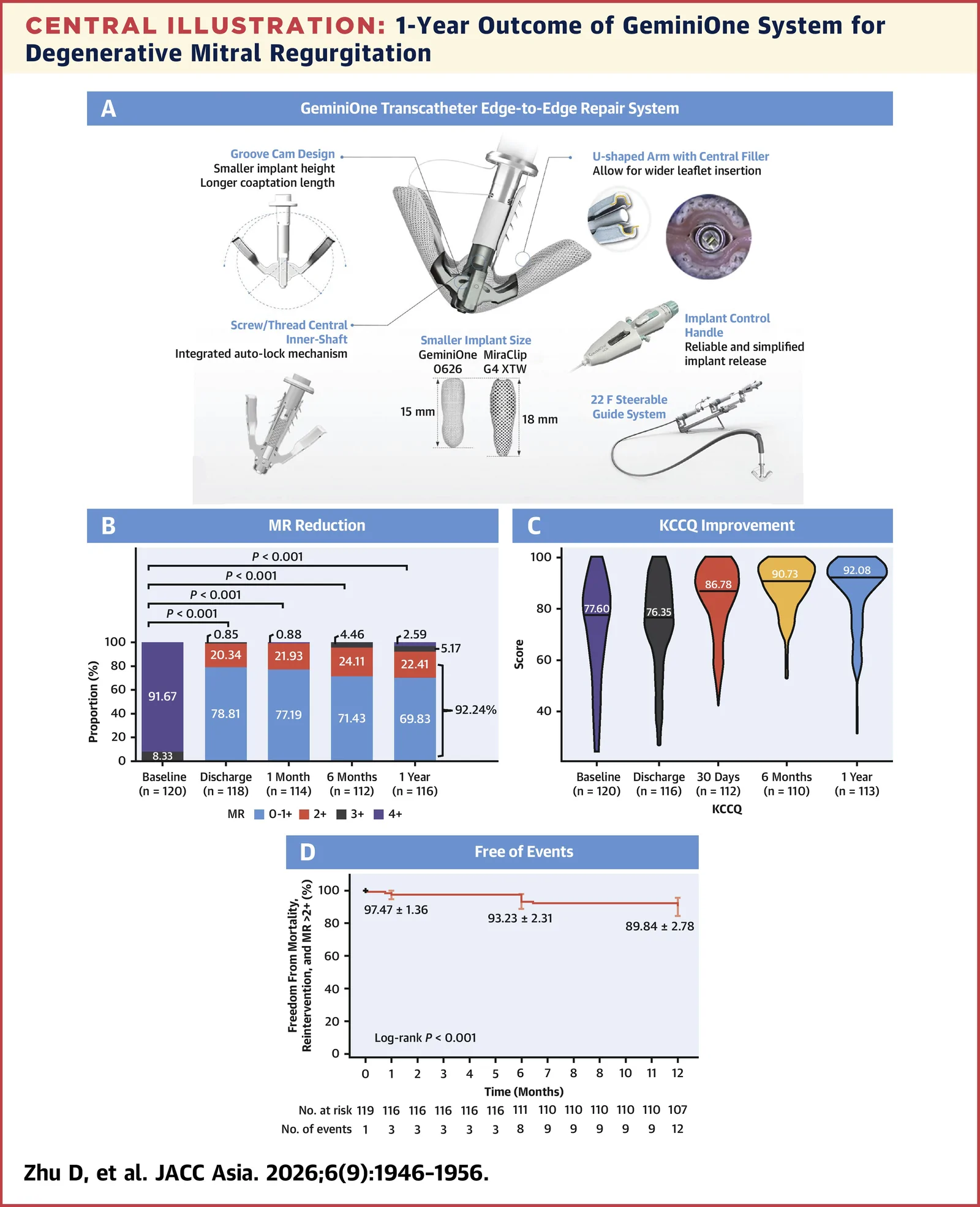
1-Year Outcome of GeminiOne System for Degenerative Mitral Regurgitation (A) Novel features of the GeminiOne transcatheter edge-to-edge repair system. (B) Mitral regurgitation (MR) reduction assessed by the core laboratroy before and after the procedure. (C) Improvement in Kansas City Cardiomyopathy Questionnaire (KCCQ) scores at 1 year, with data presented as medians. (D) The Kaplan-Meier estimates for freedom from composite rate of all-cause mortality, reintervention for mitral valve dysfunction, and MR >2+ at 1 year.

### Study limitations

This single-arm, nonrandomized study included a relatively small sample of the Chinese population, characterized by a comparatively young average age, which limits the generalizability of the findings. The follow-up period was also restricted to 1 year, preventing assessment of long-term durability and sustained clinical benefits. The study did not include patients with functional MR or complex degenerative etiologies, and the absence of a comparator TEER arm further narrowed the applicability of the results. Further large-scale randomized studies with longer follow-up are still needed to validate the safety and efficacy, as well as the durability, of the GeminiOne system in treatment of high-risk patients with DMR.

## Conclusions

In this clinical study, the GeminiOne system was safe and effective in high–surgical-risk patients suffering from moderate-to-severe DMR. The study met its primary safety and performance endpoints, showing a sustained reduction in MR along with clinically meaningful improvements in both symptoms and functional status through 1 year, comparable to established TEER devices. These benefits were accompanied by an operator-friendly design and procedural simplicity. Together, these findings support the safety and performance of the GeminiOne system.

## Funding Support and Author Disclosures

This work was supported by the Noncommunicable Chronic Diseases–National Science and Technology Major Project (2024ZD0527100/2024ZD0527500) and the program of Fuwai Yunnan Hospital, Chinese Academy of Medical Sciences (2025YFKT-ZL-01). The authors have reported that they have no relationships relevant to the contents of this paper to disclose.

## Appendix

Video 1 Desigen and Working Mechanism GeminiOne System.
