# Supplementary material for: Novel Transcatheter Edge-to-Edge Repair System for Degenerative Mitral Regurgitation: 1-Year Outcomes
Source: JACC Asia. 2026 Jun 12;6(9):1946–56. doi: 10.1016/j.jacasi.2026.04.013 (PMC13554755; doi:10.1016/j.jacasi.2026.04.013)
Supplement: Supplementary file 1 [file mmc2.docx]

**Supplementary Table S1: Inclusion and exclusion criteria**

### **Inclusion Criteria**

All of the following inclusion criteria must be met to be selected:

[1] Patients who voluntarily participate and sign the informed consent form, understand the purpose of the study, and can cooperate with the entire study process;
[2] Age≥ 18 years old;
[3] Subject has symptomatic moderate-to-severe or severe degenerative mitral regurgitation (defined as mitral regurgitatio≥3+) determined by diagnostic transthoracic echocardiography (TTE) obtained within 90 days and transesophageal echocardiography (TEE) obtained within 180 days.
[4] The patient's New York Heart Association (NYHA) cardiac function class is II., III., or ambulatory IV.;
[5] Left ventricular ejection fraction LVEF ≥ 20%;
[6] Patients judged by the multidisciplinary cardiac team to be at high surgical risk;
[7] Anatomically suitable for mitral valve repair as judged by the multidisciplinary heart team and assessed as suitable for use of this investigational device. Key points include, but are not limited to, the following:
    a) MVA≥ 4.0 cm^2^;
    b) Left ventricular end-systolic diameter LVESD ≤ 60 mm;
    c) The primary reugrgitant jet is non-commissural, if a secondary jet is present, it must be considered clinically insignificant
    d) Transseptal catheterization and femoral vein puncture are feasible.

The recommended reference criteria for high surgical risk are: STS score of ≥8 points for surgical mitral valve replacement or STS score ≥6 points for surgical mitral valve repair, or the presence of other high-risk factors, such as ≥ 2 moderate to severe frailty indicators or procedure-specific impediments or the presence of ≥2 major organ dysfunctions that cannot be improved after surgery or other surgical high-risk factors that are clearly present as judged by the independent cardiac surgeon of this study.

### **Exclusion Criteria**

Subjects who meet any of the following exclusion criteria will not be selected:

[1] Echocardiography suggests the presence of intracardiac mass, thrombus or vegetation;
[2] Presence of other severe heart valve disease requiring intervention;
[3] Leaflet anatomy may preclude GeminiOne implantation, proper device positioning on the leaflets, or prevent sufficient reduction of mitral regurgitation. This evaluation is based on transesophageal echocardiography (TEE) mitral valve evaluation within 180 days prior to subject enrollment:
    a) Insufficient mobile leaflet available for grasping with the device
    b) Lack of both primary and secondary chordal support in the grasping area.
    c) Evidence of significant calcification in the grasping area
    d) Presence of significant cleft in the grasping area
[4] Active endocarditis, pericarditis, or rheumatic heart disease; or mitral valve leaflet degeneration caused by endocarditis and rheumatic heart disease;
[5] Severe right ventricular insufficiency (such as edema of both lower extremities with symptoms such as increased jugular venous pressure and liver enlargement); or pulmonary hypertension (pulmonary artery systolic pressure PASP > 70mmHg as measured by echocardiography);
[6] Acute coronary syndrome within 4 weeks, or untreated severe coronary artery stenosis requiring revascularization;
[7] Any cardiovascular intervention, cardiac surgery, cardiac resynchronization therapy (CRT, CRT-D), implantation of a cardioverter defibrillator (ICD), etc. within 30 days; or those who plan to undergo the above surgery;
[8] Any mitral valve surgery or mitral valve transcatheter intervention; or have a left atrial appendage closure device;
[9] end-stage heart failure (ACC/AHA stage D ), or after heart transplantation, or awaiting heart transplantation;
[10] Hemodynamic instability, defined as systolic blood pressure < 90mmHg without afterload-reducing drugs with symptoms of hypoperfusion, or cardiogenic shock, or requiring vasoactive drug therapy, or requiring intra-aortic balloon recoil or any other hemodynamic support device
[11] Need for emergency or emergency surgery for any reason;
[12] The subject's femoral vein cannot accommodate a 22F catheter or has ipsilateral deep vein thrombosis, or the patient's anatomy is not suitable for transseptal puncture;
[13] Active infection needs to be treated with current antibiotic therapy; If it is a temporary disease, antibiotics must be discontinued for at least 14 days before enrollment;
[14] History of any cerebrovascular accident within 30 days; or severe symptomatic carotid artery stenosis (carotid ultrasound suggests stenosis >70%);
[15] Modified Rankin scale≥ 4 points;
[16] History of acute peptic ulcer or gastrointestinal bleeding within 3 months;
[17] Hematological cachexia, including granulocytopenia (WBC<3×10 ^9^ /L), anemia (HB< 90g/L), thrombocytopenia (PLT<50×10 ^9^/L), bleeding disorders and coagulation dysfunction diseases; or contraindications to antiplatelet or anticoagulant therapy;
[18] There are contraindications to transesophageal echocardiography; or presence of contraindications to general anesthesia;
[19] Severe chronic obstructive pulmonary disease (COPD) requiring long-term steroid use or continuous home oxygen therapy;
[20] Patients with severe hepatic and renal insufficiency (ALT, AST, creatinine greater than twice the upper limit of normal);
[21] Pregnant or lactating women, or female subjects with a birth plan within 12 months;
[22] Patients with known allergies to device components or contrast agents;
[23] Patients enrolled in another clinical study and in the follow-up period;
[24] Diseases that make treatment evaluation difficult (e.g., cancer, infection, severe metabolic diseases, mental illness, etc.); or patients with a life expectancy of less than 12 months;
[25] Patients who are judged by the investigator to have poor compliance and are unable to comply with the follow-up plan and complete the examination; or other situations in which the investigator believes that the subject is not suitable to participate in the study.

**Supplementary Table S2: Trial organization and participating sites**

| **1.** **Coordinating Investigator** Professor Hu Shengshou | | |
| --- | --- | --- |
| **2.** **Clinical study institution** | **PI** | **Initiated on** |
| 01 Fuwai Yunnan Hospital | Pan Xiangbin | 2022-11-11 |
| 02 West China Hospital Sichuan University | Chen Mao | 2023-03-09 |
| 03 The Second Xiangya Hospital of Central South University | Zhao Shenghua | 2023-02-27 |
| 04 Shanghai Chest Hospital | He Ben | 2023-06-05 |
| 05 Southwest Hospital | Zhang Zhihui | 2023-03-16 |
| 07 Mianyang Central Hospital | Luo Caidong | 2023-04-25 |
| 08 The Second Affiliated Hospital of Air Force Military University | Li Yan | 2023-05-11 |
| 13 The First Affiliated Hospital of Air Force Military University | Yang Jian | 2024-02-06 |
| 14 The First Affiliated Hospital of Nanchang University | Peng Xiaoping | 2024-03-28 |
| 15 Zhongnan Hospital of Wuhan University | Liu Jinping | 2023-03-03 |
| 18 Meizhou People's Hospital | Zhong Wei | 2023-08-15 |
| 19 Fuwai Central China Cardiovascular Hospital | Liu Yuhao | 2023-5-17 |
| 24 Fujian Medical University Union Hospital | Chen Liangwan | 2023-2-17 |
| 26 Xiamen Cardiovascular Hospital Xiamen University | Wang Yan | 2023-9-15 |
| 27 Jiangsu Province Hospital | Kong Xiangqing | 2024-02-02 |
| 30 The First Affiliated Hospital of USTC (Anhui Provincial Hospital) | Xu Jian | 2023-1-17 |
| 34 The First Affiliated Hospital, Zhejiang University School of Medicine | Ma Liang | 2023-10-20 |
| 39 Nanfang Hospital | Xiu Jiancheng | 2023-07-19 |
| 40 The Second Affiliated Hospital, Zhejiang University School of Medicine | Wang Jian'an | 2023-07-17 |
| 41 Fuwai Hospital | Pan Xiangbin | 2023-12-27 |
| 42 The First Affiliated Hospital of Bengbu Medical University | Liu Jinjun | 2023-12-26 |
| 43 Zhuzhou Central Hospital | Ouyang Fan | 2023-06-01 |
| **3. Data management and statistical analysis****:** National Center for Cardiovascular Diseases, Department of Medical Statistics | | |
| **4. Sponsor:** Peijia Medical Limited, Suzhou. | | |
| **5. Monitoring Institution:** Coremed Medtech Beijing | | |

**Supplementary Table 3: Kaplan-Meier Estimated 1- Year Event-Free Survival**

| **Event** | **Freedom from occurance Rate (95% CI)** | **Rate ± standard error** |
| --- | --- | --- |
| All-cause mortality | 97.48（94.7 to 100) | 97.48±1.35 |
| Reintervention for mitral valve dysfunction | 98.31（96.02 to 100) | 98.31±1.02 |
| MR >2+ | 91.45（86.52 to 96.66) | 91.45±2.59 |
| Heart failure hospitalization | 94（89.79 to 98.41) | 94.00±2.20 |
| Composite of all-cause mortality, reintervention for mitral valve dysfunction and MR >2+ | 89.84（84.56 to 95.46) | 89.84±2.78 |
| Composite of all-cause mortality, reintervention for mitral valve dysfunction, MR >2+ and HF rehospitalization | 84.76（78.52 to 91.5) | 84.76±3.31 |
| MAEs | 91.61（86.76 to 96.73) | 91.61±2.54 |
